# Complete Genome Sequences of Evolutionary Clade C-III Strains of *Clostridioides* (*Clostridium*) *difficile* Isolated from the Environment in Western Australia

**DOI:** 10.1128/mra.00239-23

**Published:** 2023-04-26

**Authors:** Nirajmohan Shivaperumal, Natasza M. R. Hain-Saunders, Barbara J. Chang, Thomas V. Riley, Daniel R. Knight

**Affiliations:** a School of Biomedical Sciences, The University of Western Australia, Nedlands, Western Australia, Australia; b Harry Butler Institute, Murdoch University, Murdoch, Western Australia, Australia; c Department of Microbiology, PathWest Laboratory Medicine WA, Nedlands, Western Australia, Australia; d School of Medical and Health Sciences, Edith Cowan University, Joondalup, Western Australia, Australia; University of Maryland School of Medicine

## Abstract

Clostridioides (Clostridium) difficile in the environment is thought to contribute to C. difficile infection in community settings. Here, we provide complete genome assemblies for two esculin hydrolysis-negative strains of C. difficile that were isolated from soils in Western Australia; the strains produce white colonies on chromogenic media and belong to evolutionarily divergent clade C-III.

## ANNOUNCEMENT

Clostridioides (Clostridium) difficile is an anaerobe that can cause life-threatening infectious diarrhea and colitis in humans and nonhuman animals (1). Laboratory detection of C. difficile often relies on growth on selective media such as C. difficile ChromID agar (bioMérieux, Marcy L’Etoile, France), on which C. difficile produces distinctive black colonies (2). However, C. difficile strains belonging to PCR ribotype 023 produce colorless or white colonies on ChromID agar and thus may evade laboratory detection (2). Recently, we reported several strains of toxigenic C. difficile that were cultured from the environment and that also produced white colonies on chromogenic agar (3, 4), attributable to an inability to hydrolyze esculin. Those strains belonged to clade C-III, a divergent evolutionary lineage that is not well understood (5).

Accurate phylogenetic analyses of C. difficile rely on complete reference genomes, which currently exist for all of the major C. difficile clades (clades 1 to 5, C-I, and C-II) but not C-III. Here, we provide complete circularized genomes for two esculin hydrolysis-negative strains of C. difficile (HGP05 and HGP14) from clade C-III that were isolated from garden soils in Perth, Western Australia, Australia.

C. difficile was originally isolated from soil samples using an anaerobic enrichment culture method, as described previously (4). In preparation for whole-genome sequencing (WGS), pure isolates of C. difficile were grown on blood agar for 48 h in an anaerobic chamber (with 80% N_2_, 10% CO_2_, and 10% H_2_). Total genomic DNA for both short-read (Illumina, San Diego, CA, USA) and long-read (Oxford Nanopore Technologies [ONT], Oxford, UK) sequencing was prepared using a QuickGene DNA tissue kit (Kurabo Industries, Osaka, Japan), without shearing or size selection. Short-read WGS was performed using standard Nextera Flex 150-bp paired-end libraries on a NovaSeq 6000 system (Illumina), to an average read depth of 104×. Reads were quality filtered and trimmed using Trim Galore v0.6.7 (https://github.com/FelixKrueger/TrimGalore). Long-read sequencing was performed on a MinION Mk1C system (ONT) using an R9 flow cell with a DNA-by-ligation protocol (SQK-LSK109). Base calling was performed using Guppy v4.0.11 integrated within MinKNOW v20.06.17. Filtlong v0.2.0 (https://github.com/rrwick/Filtlong) was used to filter low-quality reads (retaining the top 90% of reads), which resulted in 3.07 Gb (HGP05) and 3.09 Gb (HGP14) of sequence data. The Illumina and ONT reads were combined for hybrid genome assembly using Unicycler v0.5.0 (6) in conservative assembly mode, with multiple rounds of polishing with Pilon v1.2.4 and Racon v1.4.3 to improve contiguity. Complete circular genomes were confirmed using Bandage v0.8.1 (7) and rotated to *dnaA* using Unicycler. Genomes were annotated using the NCBI Prokaryotic Genome Annotation Pipeline (PGAP) v6.3 (8). Sequence type (ST) was determined using PubMLST (BIGSdb v1.32.0) (9). Prophages and toxin gene loci were characterized using PHASTER (https://phaster.ca) and Abricate v1.0 (https://github.com/tseemann/abricate), respectively. Default parameters were used for all software unless otherwise specified.

Information on the assembled chromosomes and extrachromosomal elements is shown in Table 1. We report the complete genome sequences of two esculin hydrolysis-negative strains of C. difficile. The white-colony phenotype may be missed in the laboratory; therefore, this data set will aid in future studies of C. difficile infection epidemiology and evolution in the community.

**TABLE 1:** Key features of new C. difficile genomes

Feature	Data for isolate:	
	HGP05	HGP14
Origin	Soil, Western Australia, 2018	Soil, Western Australia, 2018
PCR ribotype	125	QX597
ST	848 (clade C-III)	632 (clade C-III)
Toxin gene profile^*a*^	A^−^ B^−^ CDT^−^	A^+^ B^−^ CDT^−^
Chromosome		
GenBank accession no.	CP103977	CP103804
Size (bp)	4,107,441	4,197,547
No. of contigs	1	1
GC content (%)	28.68	28.71
No. of coding sequences	3,693	3,926
No. of tRNAs	91	90
No. of rRNAs	35	35
No. of CRISPRs^*b*^	7	3
Prophages	ΦCDMH1, ΦMMP01, ΦCDHM19	ΦMMPO3, ΦMMPO1, ΦCDHM19
Extrachromosomal element size (bp) (GenBank accession no.)		
Novel plasmid	5,997 (CP103978)^*c*^	
Novel phage-like element	63,696 (CP103976)^*d*^	66,910 (CP103805),^*e*^ 35,072 (CP103806)^*f*^
Illumina reads		
Total no. of reads (trimmed)	1,517,442	1,470,894
Avg read length (trimmed) (bp)	150	150
SRA accession no.	SRR16474727	SRR16474718
ONT reads		
Total no. of reads (filtered)	1,846,067	3,280,985
Avg read length (filtered) bp)	1,800	1,000
Base-called *N*_50_ (bp)	7,450	4,520
SRA accession no.	SRR23755003	SRR23755002

a Toxin profile: presence/absence of full-length *tcdA* (A), *tcdB* (pathogenicity locus) (B), and binary toxin *cdtA/B* (binary toxin locus) (CDT).

b CRISPRs, clusters of regularly interspaced short palindromic repeats.

c Novel, circularized plasmid showing homology with C. difficile pCD6 plasmid (GenBank accession number NC_005326) (85% sequence identity across 47% of the pCD6 genome).

d Novel, circularized phage-like element containing a 30.2-kb region showing homology with C. difficile phage ΦCDHM19 (GenBank accession number NC_028996) (75% sequence identity across 18% of the ΦCDHM19 genome).

e Novel, circularized phage-like element containing a 22.4-kb region showing homology with C. difficile phage ΦCDHM19 (GenBank accession number NC_028996) (75% sequence identity across 15% of the ΦCDHM19 genome). The ΦCDHM19-like regions from HGP05 and HGP14 show significant homology (96% sequence identity across 70% of the sites).

f Novel, circularized phage-like element containing a 34.0-kb region showing homology with ΦCD111 (GenBank accession number NC_028905) (89% sequence identity across 66% of the ΦCD111 genome).

### Data availability.

Complete genome assemblies for HGP05 and HGP14 are available in GenBank under BioProject accession number PRJNA772357 (accession numbers CP103976, CP103977, and CP103978 and accession numbers CP103804, CP103805, and CP103806, respectively). Sequence read data are available in the NCBI Sequence Read Archive (SRA) under BioProject accession number PRJNA772357 (Illumina reads: SRA accession numbers SRR16474727 [HGP05] and SRR16474718 [HGP14]; Nanopore reads: SRA accession numbers SRR23755003 [HGP05] and SRR23755002 [HGP14]).
